# Statistical significance of variables driving systematic variation in high-dimensional data

**DOI:** 10.1093/bioinformatics/btu674

**Published:** 2014-10-21

**Authors:** Neo Christopher Chung, John D. Storey

**Affiliations:** ^1^Lewis-Sigler Institute for Integrative Genomics and ^2^Department of Molecular Biology, Princeton University, Princeton, NJ 08544, USA

## Abstract

**Motivation**: There are a number of well-established methods such as principal component analysis (PCA) for automatically capturing systematic variation due to latent variables in large-scale genomic data. PCA and related methods may directly provide a quantitative characterization of a complex biological variable that is otherwise difficult to precisely define or model. An unsolved problem in this context is how to systematically identify the genomic variables that are drivers of systematic variation captured by PCA. Principal components (PCs) (and other estimates of systematic variation) are directly constructed from the genomic variables themselves, making measures of statistical significance artificially inflated when using conventional methods due to over-fitting.

**Results**: We introduce a new approach called the *jackstraw* that allows one to accurately identify genomic variables that are statistically significantly associated with any subset or linear combination of PCs. The proposed method can greatly simplify complex significance testing problems encountered in genomics and can be used to identify the genomic variables significantly associated with latent variables. Using simulation, we demonstrate that our method attains accurate measures of statistical significance over a range of relevant scenarios. We consider yeast cell-cycle gene expression data, and show that the proposed method can be used to straightforwardly identify genes that are cell-cycle regulated with an accurate measure of statistical significance. We also analyze gene expression data from post-trauma patients, allowing the gene expression data to provide a molecularly driven phenotype. Using our method, we find a greater enrichment for inflammatory-related gene sets compared to the original analysis that uses a clinically defined, although likely imprecise, phenotype. The proposed method provides a useful bridge between large-scale quantifications of systematic variation and gene-level significance analyses.

**Availability and implementation**: An R software package, called jackstraw, is available in CRAN.

**Contact**: jstorey@princeton.edu

## 1 INTRODUCTION

**Fig. 1.**
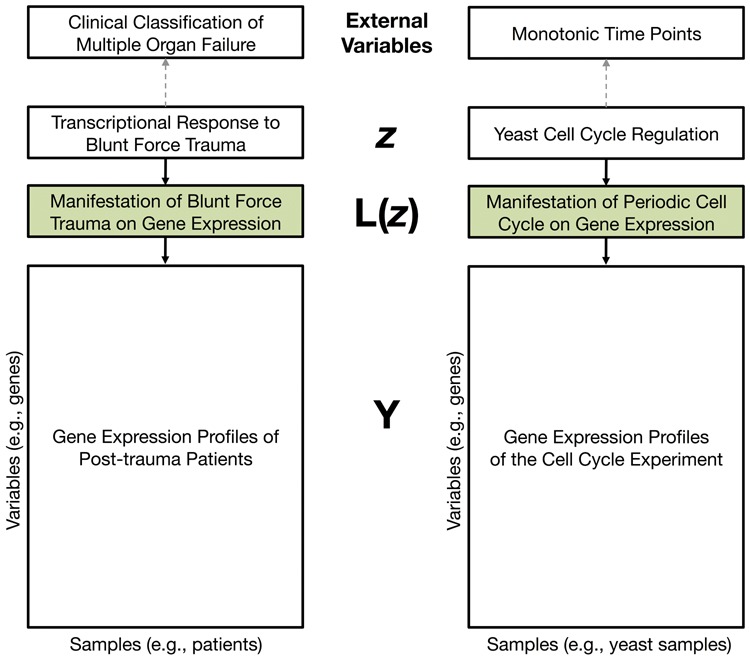
Illustration of systematic variation genomic data due to latent variables. Complex biological variables, such as clinical subtypes and cell-cycle regulation, may be difficult to define, measure, or model. Instead, we can characterize the manifestation of latent variables, L(z), directly from high-dimensional genomic data using PCA and related methods. The proposed method calculates the statistical significance of associations between variables in **Y** and estimates of **L**, while accounting for over-fitting due to the fact that **L** must be estimated from **Y**

Latent variable models play an important role in understanding variation in genomic data (Leek and Storey, 2007; Price *et al.*, 2006). They are particularly useful for characterizing systematic variation in genomic data whose variable representation is unobserved or imprecisely known (Fig. 1). Principal component analysis (PCA) has proven to be an especially informative method for capturing quantitative signatures of latent variables in genomic data, and it is in widespread use across a range of applications. For example, PCA has been successfully applied to uncover the systematic variation in gene expression (Alter *et al.*, 2000; Holter *et al.*, 2000; Raychaudhuri *et al.*, 2000), estimate structure in population genetics (Price *et al.*, 2006; Zhu *et al.*, 2002), and account for dependence in multiple hypothesis testing (Leek and Storey, 2007, 2008). Generally, principal components (PCs) can be thought of as estimates of unobserved manifestation of latent variables; they are constructed by aggregating variation across thousands or more genomic variables (Jolliffe, 2002). What is missing from this highly successful system is a method to precisely identify which genomic variables are the statistically significant drivers of the PCs in genomic data, which in turn identifies the genomic variables associated with the unobserved latent variables.

In a typical application of PCA to genomic data, all variables will have non-zero loadings, meaning that they all make some contribution to the construction of PCs. We refer to genomic variables as the high-dimensional variables considered in a genomics study such as genes, array probe sets, or genetic loci. In some cases, when many (or most) of these contributions are forcibly set to zero, similar PCs nevertheless emerge. Methods have been proposed to induce sparsity in the loadings, for example, with a lasso penalized PCA or a Bayesian prior (Engelhardt and Stephens, 2010; Jolliffe *et al.*, 2003;Witten *et al.*, 2009; Zou *et al.*, 2006). Methods have also been developed to consider uncertainty in PCA expansions (Goldsmith *et al.*, 2013). Various formulations of statistical significance have been considered previously in the context of PCA. These have usually been focused on scenarios where the number of observations is substantially larger than the number of variables, significance is measured in terms of a completely unstructured data matrix where all variables are mutually independent, or the goal is to only determine the number of significant PCs (Anderson, 1963; Buja and Eyuboglu, 1992; Girshick, 1939; Johnstone, 2001; Linting *et al.*, 2011; Peres-Neto *et al.*, 2003; Tracy and Widom, 1996; Timmerman *et al.*, 2010). The problem we consider here differs from those scenarios.

Our goal is not a minimal representation of a PCA; we would like instead to develop a strategy that accurately identifies which genomic variables are truly associated with systematic variation of interest. This can be phrased in statistical terminology as developing a significance test for associations between genomic variables and a given set, subset, or linear combination of PCs estimated from genomic data. We introduce a new resampling approach, which we call the *jackstraw*, to rigorously identify the genomic variables associated with PCs of interest, as well as subsets and rotations of PCs of interest. Our approach is capable of obtaining the empirical null distribution of association statistics (e.g. *F*-statistics) and applying these to the observed association statistics between genomic features and PCs to obtain valid statistical significance measures. Succinctly, new PCs are computed from a dataset with a few independently permuted variables, which become tractable ‘synthetic’ null variables. The association statistics between newly computed PCs and synthetic null variables serve as empirical null statistics, accounting for the measurement error and over-fitting of PCA.

As an application, we consider the problem of identifying genes whose expression is cell-cycle regulated. In this case, there are infinitely many theoretical curves that would represent ‘cell-cycle regulation’ to the point where a standard statistical analysis involves an unwieldy ‘composite null hypothesis’ (Lehmann, 1997). We identify the few realized patterns of cell-cycle regulated gene expression through PCA and we are able to directly test whether each gene is associated with these using the proposed approach. As another application, we analyzed observational gene expression profiles of blunt-force trauma patients (Desai *et al.*, 2011), whose post-trauma inflammatory responses are difficult to be quantified using conventional means. When the clinical phenotype of interest cannot be precisely measured and modeled, we may estimate it directly from genomic data itself. We identify genes driving systematic variation in gene expression of post-trauma patients and demonstrate that our analysis is biologically richer than the original analysis (Desai *et al.*, 2011).

PCA has direct connections to independent component analysis (ICA; Hastie *et al.*, 2011) and *K*-means clustering (Ding and He, 2004; Zha *et al.*, 2001). Therefore, the methods we propose are likely applicable to those models as well. Furthermore, this approach has potential generalizations to a much broader class of clustering and latent variable methods that all seek to capture systematic variation.

## 2 STATISTICAL MODEL AND APPROACH

Consider an *m* × *n* row-wise mean-centered expression data matrix **Y** with *m* observed variables measured over *n* observations (m≫n). **Y** may contain systematic variation across the variables from an arbitrarily complex function of latent variables ***z***. We may calculate the expected influence of the latent variables on **Y** by E[Y|z], and then write Y=E[Y|z]+E, where **E** is defined as Y−E[Y|z]. There exists a *r* × *n* matrix, called L(z), that is a row basis for E[Y|z], where r≤n (Leek and Storey, 2007, 2008). This low-dimensional matrix L(z) can be thought of as the manifestation of the latent variables in the genomic data. As illustrated in Figure 1, this conditional factor model is common for biomedical and genomic data (Leek, 2010). Since ***z*** is never directly observed or used in the model, we will abbreviate L(z) as **L**. This yields the model

(1) Y=BL+E

where B is a *m* × *r* matrix of unknown parameters of interest. The $i^{\text{th}}$ row of B, which we write as $b_{i}$, quantifies the relationship between the latent variable basis **L** and genomic variable $y_{i}$. This model (1) is schematized in Supplementary Material, Figure S1.

The PCs of **Y** may be calculated by taking the singular value decomposition (SVD) of **Y**. This yields $Y=UDV^{T}$ where U is a *m* × *n* orthonormal matrix, D is a *n* × *n* diagonal matrix and V is a *n* × *n* orthonormal matrix. The diagonal elements in D are the *n* singular values, which are in a decreasing order of magnitude. The rows of $V^{T}$ are the right singular vectors, with corresponding singular values in D. PCs are then the rows of $DV^{T}$, where the $i^{\text{th}}$ PC is found in the $i^{\text{th}}$ row of $DV^{T}$. The columns of U are considered to be the loadings of their respective PCs.

Suppose that the row-space of **L** has dimension *r*. The top *r* PCs may then be used to estimate the row basis for **L** (Jolliffe, 2002). Specifically, under a mild set of assumptions, it has been shown that as m→∞, the top *r* PCs of **Y** converge with probability 1 to a matrix whose row space is equivalent to that of **L** (Leek, 2010). For our estimation purposes, we only need to consider the $V^{T}$ matrix since this captures the row-space. We would therefore estimate **L** by simply obtaining the top *r* right singular vectors, which we denote by $V_{r}^{T}$.

**Fig. 2.**
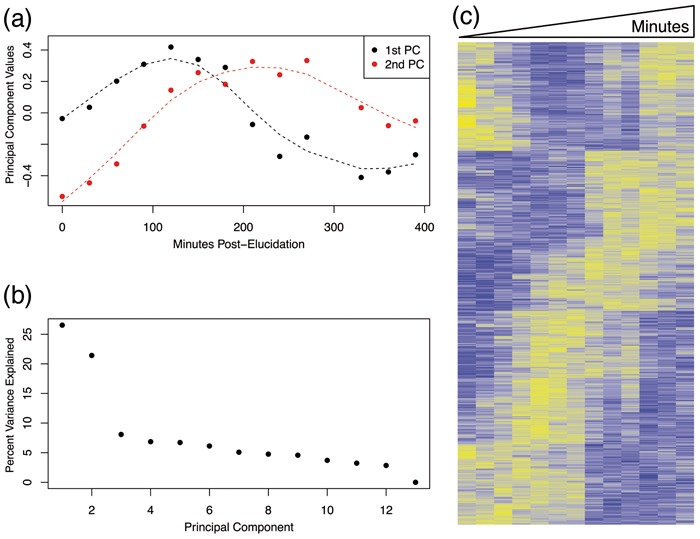
Identification of yeast genes associated with the cell-cycle regulation. (a) The top two PCs of gene expression measured over time in a population of yeast whose cell cycles have been synchronized by elutriation; these PCs appear to capture cell-cycle regulation patterns (Spellman et al., 1998). The dashed lines are natural cubic smoothing splines fit to each PC, respectively (with 5 degrees of freedom). (b) The percent variance explained by PCs shows that the top two PCs capture 48% of the total variance in the data. (c) Hierarchical clustering of expression levels of genes significantly associated with the top two PCs at FDR≤1%, where rows are genes and columns are time points. Hierarchical clustering was applied to this subset of 2998 genes

Let’s now consider a concrete example of $z,\;L,\;V_{r}^{T}$, and the ultimate inference goal. Spellman *et al.* (1998) carried out a gene expression study to identify cell-cycle regulated genes of *Saccharomyces cerevisiae* (Fig. 2). In this experiment, *m* = 5981 genes’ expression values were originally measured over *n* = 14 time points in a culture of yeast cells whose cell cycles had been synchronized. (Note that an inspection of the 14 microarrays from Spellman *et al.* (1998) reveals an aberrant gene expression profile from 300-min, so we removed this array in our analysis—see Supplementary Figure S2.) Here, ***z*** is the latent variable that represents the dynamic gene expression regulatory program over the yeast cell cycle. **L** is the manifested influence of ***z*** on the observed scale of gene expression measurements (Fig. 1). The ordered time points themselves do not capture the underlying cell-cycle regulation, and it is, therefore, not clear how to *a priori* accurately model **L**. If **L** were directly observed, then we could identify which genes are cell-cycle regulated by performing a significance test of $H_{0}:b_{i}=0$ versus H1:bi=0 for each gene *i*.

However, since **L** is not observed, we can instead perform the analogous association test using $V_{r}^{T}$. Figure 2(a) shows the first two PCs of **Y**, where it can be seen that these capture systematic variation that resembles cell-cycle regulation. (It should be noted that the remaining PCs, three and higher, do not appear to capture systematic variation of interest.) Since the row-spaces of **L** and $V_{r}^{T}$ (*r* = 2) are theoretically close (Leek, 2010), we can instead use the model

(2) $$Y=\Gamma V_{r}^{T}+E^{\prime },$$

where Γ is a *m* × *r* matrix of unknown coefficients. We would then perform a significance test of $H_{0}:\gamma_{i}=0$ versus $H_{1}:\gamma_{i}\neq 0$ for each gene *i*.

Note that if $V_{r}^{T}\to L$ in row-space as m→∞, then these two hypothesis tests would be asymptotically (in the number of variables) equivalent. However, for fixed *m*, they are not equivalent. There are two main issues: (i) $V_{r}^{T}$ is a noisy estimate of **L**; (ii) $V_{r}^{T}$ is itself a function of **Y**, so hypothesis testing on $Y=\Gamma V_{r}^{T}+E^{\prime }$ results in an anti-conservative bias due to overfitting. Our proposed method deals with problem (ii) by accounting for the over-fitting that is intrinsic to performing hypothesis testing on model (2). The numerical results in this article are carried out so that we generate the data from model (1) and evaluate the accuracy of the significance based on the truth from model (1). Therefore, our thorough simulations provide evidence that the proposed method accounts for both issues (i) and (ii).

## 3 PROPOSED ALGORITHMS

**Fig. 3.**
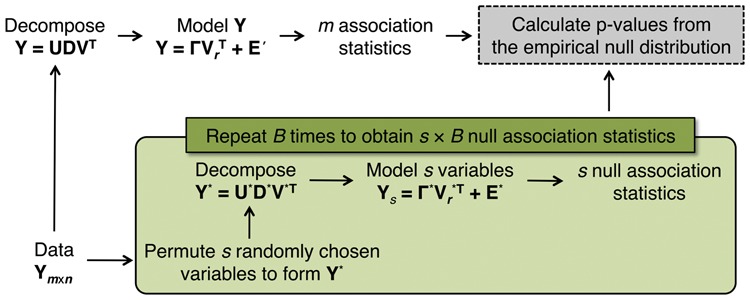
A schematic of the general steps of the proposed algorithm to calculate the statistical significance of associations between variables (rows in **Y**) and their top *r* PCs ($V_{r}^{T}$). By independently permuting a small number (*s*) of variables and recalculating the PCs, we generate tractable “synthetic” null variables while preserving the overall systematic variation. Association statistics between the *s* synthetic null variables in $Y^{*}$ and $V_{r}^{*T}$ form the empirical null distribution, automatically taking account over-fitting intrinsic to testing for associations between a set of observed variables and their PCs

We have developed a resampling method (Fig. 3) to obtain accurate statistical significance measures of the associations between observed variables and their PCs, accounting for the over-fitting characteristics due to computation of PCs from the same set of observed variables. The proposed algorithm replaces a small number *s* (s≪m) of observed variables with independently permuted ‘synthetic’ null variables, while preserving the overall systematic variation in the data. Note that the jackstraw disrupts the systematic variation among the randomly chosen *s* rows by applying independently generated permutation mappings. We denote the new matrix with the *s* synthetic null variables replacing their original values as $Y_{m\times n}^{*}$. This is simply the original matrix **Y** with the *s* rows of **Y** replaced by independently permuted versions. On each permutation dataset $Y^{*}$, we calculate association statistics for each synthetic null variable, exactly as was done on the original data. We carry this out *B* times, effectively creating *B* sets of permutation statistics. The association statistics calculated on **Y** are then compared to the association statistics calculated on only the *s* synthetic null rows of $Y^{*}$ to obtain statistical significance measures.

**Algorithm to Calculate Significance of Variables Associated with PCs**

1. Obtain *r* PCs of interest, $V_{r}^{T}$ by applying SVD to the row-wise mean-centered matrix $Y_{m\times n}=UDV^{T}$.
2. Calculate *m* observed *F*-statistics $F_{1},\ldots ,F_{m}$, testing $H_{0}:\gamma_{i}=0$ versus $H_{1}:\gamma_{i}\neq 0$ from model (2).
3. Randomly select and permute *s* rows of $Y_{m\times n}$, resulting in $Y_{m\times n}^{*}$.
4. Obtain $V_{r}^{*T}$ from SVD applied to $Y^{*}=U^{*}D^{*}V^{*T}$.
5. Calculate null *F*-statistics $F_{1}^{0b},\ldots ,F_{s}^{0b}$ from the *s* synthetic null rows of $Y^{*}$ as in step 2, where $V_{r}^{T}$ is replaced with $V_{r}^{*T}$.
6. Repeat steps 3–5 b=1,…,B times to obtain a total *s* × *B* of null *F*-statistics.
7. Compute the *P* value for variable *i* (i=1,…,m) by:

$$p_{i}=\frac{\#\{F_{j}^{0b}\geq F_{i};j=1,\ldots ,s,b=1,\ldots ,B\}}{s\times B}$$

8. Identify statistically significant tests based on the *P* values $p_{1},p_{2},\ldots ,$ *p_m_* (e.g. using false discovery rates).

We call this approach the *jackstraw* for the following reason. By permuting a relatively small amount of observed variables in the original matrix, the underlying systematic variation due to latent variables is preserved as a whole. This makes the PCs of $Y^{*}$ almost identical to the PCs of the original data, **Y**, up to variation due to over-fitting of the noise. Replacing *s* variables with null versions is reminiscent of the game of jackstraws where the goal is to remove one stick at a time from a structured set of sticks without disrupting the overall structure of the sticks. Since the overall structure of **Y** is preserved in $Y^{*}$, we know that the level of associations between these synthetic null variables and the top *r* PCs is purely due to the over-fitting nature of PCA. From these synthetic null statistics, we can, therefore, capture and adjust for the over-fitting among the original statistics.

A balance between the number of resampling iterations *B* and the number of synthetic null variables *s* is relevant to the speed of the algorithm and the accuracy of the resulting *P* values. In each resampling iteration, *s* determines the number of estimated null statistics, so to get the same resolution of a particular empirical null distribution (*s* × *B* total null statistics), *B* must increase proportionally with a decreasing *s*. Suppose we fix the total number of null statistics *s* × *B* that are generated (e.g. s×B=10 000). One extreme is to set *s* = 1 and *B* = 10 000, where the accuracy of the *P* values is maximized while the algorithm is the least efficient. However, setting *s* = 100 and *B* = 100 yields the same number of null statistics; this configuration would lead to a savings in computational time while it may result in slightly more conservative *P* values. The number of true null variables in $Y^{*}$ is always greater than or equal to the number of true null variables in the original matrix **Y**. Therefore, an increase of *s* in the proposed algorithm may lead to a greater over-fitting into the noise of $Y^{*}$ relative to the over-fitting in **Y**, resulting in conservative estimates of significance. Due to this favorable trade-off between *s* and *B*, the proposed algorithm is guarded against anti-conservative bias.

The hypothesis test $H_{0}:\gamma_{i}=0$ versus $H_{1}:\gamma_{i}\neq 0$ applied to model (2) may be generalized to performing the test on subspaces spanned by the PCs, shown in Supplementary Material. This generalization allows one to perform the association tests on a subset of PCs, while adjusting for other PCs. It also allows for one to consider rotations of $V_{r}^{T}$ and projections of $V_{r}^{T}$ onto relevant subspaces. For example, it may be possible to rotate the PCs to obtain ‘independent components’ from ICA (Hastie *et al.*, 2011) and then perform our algorithm on any desired subset of the independent components. Note that when a subset of $V_{r}^{T}$ is considered, the largest *r* eigenvalues corresponding to the top *r* PCs must be sufficiently distinguished to ensure their stability (Ng *et al.*, 2001).

## 4 RESULTS

We evaluated the proposed method on simulated data so that we could directly assess its accuracy, and we also applied the method to two genomic datasets to demonstrate its utility in practice.

### 4.1 Simulation studies

Through a set of simulation studies, we demonstrated that the proposed method is able to accurately estimate the statistical significance of associations between the latent variable basis **L** and observed variables $y_{i}$ (where i=1…m). The data in our simulation studies were generated from model (1) Y=BL+E, where variables $y_{i}$ corresponding to $b_{i}=0$ are, by definition, the ‘null variables’ not associated with **L** (Supplementary Fig. S1). The accuracy of our approach is evaluated by performing *m* hypothesis tests using the proposed algorithm (where only **Y** is observed) and assessing whether the joint distribution of *P* values corresponding to the null variables is correctly behaved.

#### 4.1.1 The joint null criterion

We used the ‘joint null criterion’ of Leek and Storey (2011) to assess whether the set of *P* values corresponding to the null variables follow the desired joint distribution (Supplementary Fig. S3). When testing a single hypothesis, a valid procedure generates null *P* values that are distributed uniformly between 0 and 1. For multiple hypothesis tests, the goal is that the set of null *P* values produced by a method satisfies the *joint null criterion*, which means their joint distribution is equivalent to a set of i.i.d. observations from the Uniform(0,1) (Leek and Storey, 2011). Verifying that the proposed method satisfies the joint null criterion not only demonstrates that the method accounts for the over-fitting inherent in methods such as PCA, but also verifies that our approach to calculating the *P* value for each variable *i* is valid, which uses the set of *s* × *B* synthetic null statistics that have been pooled across variables. Leek and Storey (2011) prove that when the joint null criterion holds, then a large body of multiple testing procedures (such as the standard false discovery rate procedures) control their respective error measure.

There are two ways in which we measured deviations from the Uniform(0,1) joint null criterion. The first is via a two-sided Kolmogorov–Smirnov test (KS test), which detects any deviation; the second is a one-sided KS test, which detects anti-conservative deviations where the null *P* values are skewed towards zero. Anti-conservative deviations will occur when a method does not properly take into account the fact that the association statistics are formed between the variables and PCs (which have been built from the variables themselves), leading to over-fitting and anti-conservative *P* values. Evaluation of the joint null criterion works by simulating many datasets (corresponding to independently repeated studies) from a given data generating process (Supplementary Fig. S3). The joint behavior of the null *P* values is then evaluated among these.

We considered 16 simulation scenarios, described below. For a given scenario, we simulated 500 independent studies and calculated 500 KS test *P* values, each of which is based on the set of null *P* values from its respective study. In other words, for 500 simulation datasets per scenario, 500 KS test *P* values are calculated to measure deviations from the Uniform(0,1); a second application of the KS test is then performed on these 500 KS *P* values to assess whether any anti-conservative deviation from the Uniform(0,1) among these studies has occurred (Supplementary Fig. S3). If the statistical method being evaluated provides accurate measures of statistical significance, the collection of double KS test *P* values must be distributed Uniform(0,1). This guards against any single simulated dataset leading one to an incorrect conclusion by chance. This technique is the ‘double KS test’ introduced by Leek and Storey (2011).

Overall, we demonstrate that our proposed method provides accurate measures of statistical significance of the associations between variables and the latent variables, when the latent variables themselves are directly estimated from the data via PCA. At the same time, we show that the conventional method does not provide accurate statistical significance measures.

#### 4.1.2 Simulation scenarios and results

**Fig. 4.**
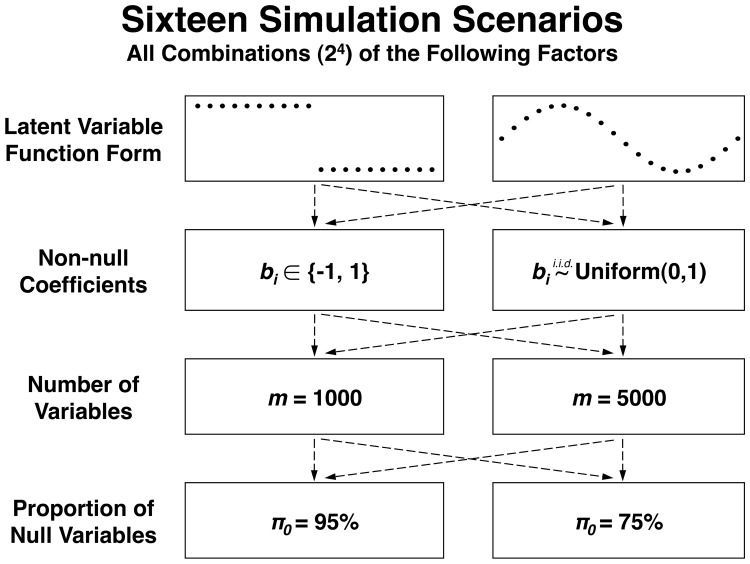
Sixteen simulation scenarios generated by combining four design factors. To assess the statistical accuracy of the conventional *F*-test and the proposed method, we simulated 500 independent studies for each scenario, and assessed statistical accuracy according to the “joint null criterion” (Leek and Storey, 2011). For the $b_{i}\in \{-1,1\}$ scenarios, non-null coefficients were set to either -1 or 1 with a probability of 0.5. For a given simulation study, a valid statistical testing procedure must yield a set of null *P* values that are jointly distributed Uniform(0,1). We use a KS test to identify deviations from the Uniform(0,1) distribution. Supplementary Material, Figure S3 provides a detailed overview of the evaluation pipeline

We constructed 16 simulation scenarios representing a wide range of configurations of signal and noise (Fig. 4), with 500 independent studies simulated from each. Let us first consider one of the simpler scenarios in detail. Model (1) is used to generate the data. In this particular scenario, we have *m* = 1000, *n* = 20, *r* = 1 and

$$L=\sqrt{\frac{n-1}{n}}(1,1,1,1,1,1,1,1,1,1,\text{-}1,\text{-}1,\text{-}1,\text{-}1,\text{-}1,\text{-}1,\text{-}1,\text{-}1,\text{-}1,\text{-}1),$$

a dichotomous mean shift resembling differential expression between the first 10 observations and the second 10 observations. (The factor $\sqrt{\frac{n-1}{n}}$ is to give **L** unit variance.) For 95% of the variables, we set *b_i_* = 0, implying they are null variables; we parameterize this proportion by $\pi_{0}=0.95$. The other 50 non-null variables were simulated such that $b_{i}\overset{\text{i.i.d}}{∼}$ Uniform(0,1). The noise terms are simulated as $e_{ij}\overset{\text{i.i.d}}{∼}$ Normal(0,1). The data for variable *i* are thus simulated according to $y_{i}=b_{i}L+e_{i}$.

**Fig. 5.**
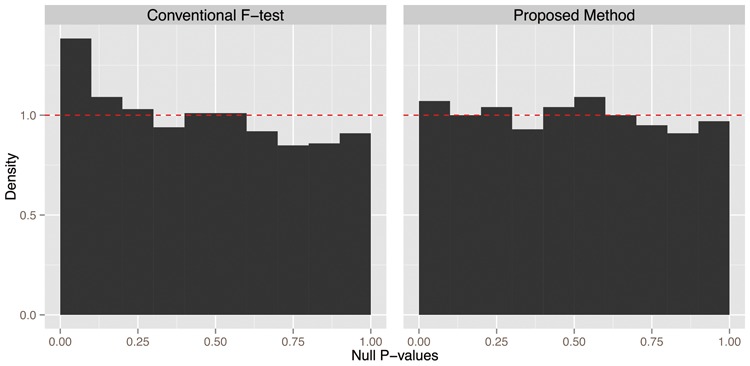
Evaluation of significance measures of associations between variables and their PCs by comparing true null *P* values and the Uniform(0,1) distribution. (a) The conventional *F*-test results in anti-conservative *P* values, as demonstrated by null *P* values being skewed towards 0. (b) The proposed method produces null *P* values distributed Uniform(0,1). The dashed line shows the Uniform(0,1) density function

For a given simulated dataset, we tested for the associations between the observed variables and the latent variables by forming association statistics between the observed $y_{1},y_{2},\ldots ,y_{m}$ and their collective PC, $V_{r}^{T}$ (*r* = 1). We calculated *P* values using both the conventional *F* test and the proposed method with *s* = 50 synthetic null variables (Fig. 5). Over 500 simulated datasets, the conventional *F* test resulted in 500 one-sided KS *P* values that exhibit a strong anti-conservative bias with a double KS *P* value of $=9.71\times 10^{-196}$ (Supplementary Fig. S4, black points). Conversely, the proposed method correctly calculates null *P* values, by accounting for the over-fitted measurement error in PCA, with a double KS *P* value of 0.502 (Supplementary Fig. S4, orange points). Alternatively, a comparison of estimated versus true FDR demonstrates an appropriate adjustment for over-fitting in the jackstraw method (Supplementary Fig. S5). Note that the classification of null *P* values is based on the true association status from the population-level data generating distribution from model (1), not based on model (2) or on the observed loadings from the PCA.

**Fig. 6.**
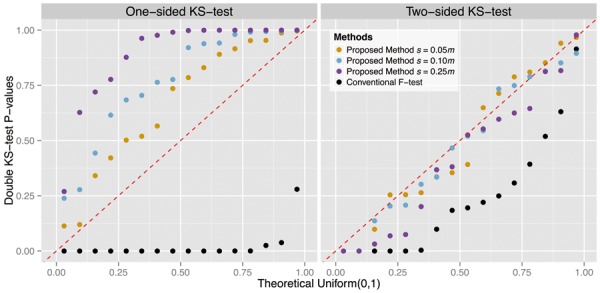
QQ-plots of double KS test *P* values from 16 simulation scenarios versus the Uniform(0,1) distribution. For each of 500 independent studies per scenario, we tested for deviation of null *P* values from Uniform(0,1), resulting in 500 KS test *P* values for each scenario. An individual point in the QQ-plot represents a double KS test *P* value for one scenario, comparing its 500 KS test *P* values to Uniform(0,1). On the left panel, the systematic downward displacement of 16 black points indicates an anti-conservative bias of the conventional *F*-test. In contrast, the proposed method produces null *P* values that are not anti-conservative. On the right panel, a set of 16 points are below the diagonal red line if the joint null distribution deviates from the Uniform(0,1) distribution. The proposed method adjusts for over-fitting of PCA and produces accurate estimates of association significance

We carried out analogous analyses on 15 more simulation scenarios, detailed in Fig. 4. We used all possible combinations of the following: (1) either dichotomous or sinusoidal functions for **L**; (2) the parameters B were simulated from either a Bernoulli or Uniform distribution; (3) *m* = 1000 or *m* = 5000 variables; and (4) the proportion of true null variables set to either $\pi_{0}=0.75$ or $\pi_{0}=0.95$. The proposed method was applied with s=0.05m, 0.10m, and 0.25m to study the impact of the choice of the number of synthetic null variables. For each scenario, we applied the joint null criterion double KS evaluation (Supplementary Fig. S3), using 500 simulated data sets. The conventional *F* test method consistently produced anti-conservative null *P* values, while the proposed method yielded accurately distributed null *P* values (Fig. 6).

In these simulations, we found that the proposed method tended to produce more conservative null *P* values as *s* increased (Fig. 6). The explanation for this is that inclusion of a larger number of synthetic null variables leads to a greater over-fitting of PCA to the noise, which in turn yields a conservative empirical null distribution formed by the synthetic null statistics. We, therefore, identified a trade-off between computational speed and how conservative the calculated *P* values are in the choice of *s*. We note, however, that the null *P* values were never observed to be prohibitively conservative in that the power became unreasonably diminished. In practice, the user has the option to lower the value of *s* to minimize this, at the cost of greater computation.

We note that we also investigated a delete-*s* version of the jackstraw, which draws on ideas from our proposed method, which one could call the permute-*s* jackstraw. However, this implementation did not produce valid null *P* values (Supplementary Material).

#### 4.1.3 Testing for associations on subsets of PCs

We have generalized the proposed method to be able to test for associations on any subset of the top *r* PCs, while adjusting for the remaining PCs among the top *r*. Here, we demonstrate that the proposed method can identify variables driving a chosen subset of PCs of interest, $V_{r_{1}}^{T}$, while adjusting for the remaining of the top *r* PCs which are not of interest, $V_{r_{0}}^{T}$, where $r_{0}+r_{1}=r$. Based on model (1), we simulated data with *m* = 1000, *n* = 20, *r* = 2 and

$$L_{1}=\sqrt{\frac{n-1}{n}}(1,1,1,1,1,1,1,1,1,1,\text{-}1,\text{-}1,\text{-}1,\text{-}1,\text{-}1,\text{-}1,\text{-}1,\text{-}1,\text{-}1,\text{-}1),$$

$$L_{2}=\sqrt{\frac{n-1}{n}}(1,1,1,1,1,\text{-}1,\text{-}1,\text{-}1,\text{-}1,\text{-}1,1,1,1,1,1,\text{-}1,\text{-}1,\text{-}1,\text{-}1,\text{-}1).$$

$L_{1}$ and $L_{2}$ are truly associated with 100 variables and 60 variables, respectively. Among these, 40 variables that are truly associated with both $L_{1}$ and $L_{2}$. We generated the noise term as $e_{ij}\overset{\text{i.i.d}}{∼}$ Normal(0,1). We set *r* = 2 and tested for associations with the first PC while adjusting for the second PC. Note that the first PC effectively captured the signal from the first latent variable. In this case, the null variables were defined to be 900 variables associated with either only the second latent variable or no latent variable. The conventional *F* test resulted in an anti-conservative bias among the null *P* values, with a double KS test *P* value of $8.73\times 10^{-20}$, while the proposed method produced a correct joint null *P* value distribution with a double KS test *P* value of 0.352 (Supplementary Fig. S6).

We performed a similar simulation with *r* = 5 true underlying latent variables and also studied the result of setting *r* to be too small or too large in model (2). For *m* = 1000 variables and *n* = 20 observations ($\pi_{0}=0.75$), we simulated *r* = 5 latent variables simulated from one of each of the following distributions: a randomized dichotomous variable, Normal(0,1), Uniform(0,1), Bin(2, 0.5), and Normal(0,0.25). We applied the jackstraw algorithm with s=0.1m and the conventional *F* test to the simulated data with $\hat{r}=1,3,5,7,9$ used in model (2). To detect an anti-conservative bias, we applied a one-sided KS-test on *P* values corresponding to the true null variables as done above. Since there exist in truth *r* = 5 latent variables, the results with $\hat{r}=1,3$ and $\hat{r}=7,9$ demonstrate the operating characteristics when the number of PCs is under- or over-specified, respectively. We found that the jackstraw method resulted in valid null *P* values while the conventional test did not (Supplementary Fig. S7).

### 4.2 Application to gene expression studies

Typically, genomic variables are tested for the associations with external variables, which are measured independently of genomic profiling technology, such as disease status, treatment labels, or time points. However, external variables may be imprecise or inaccurate due to poor understanding of the biology or technological limitations; sometimes the external variables of interest may not be capable of being measured at all. For example, in a cancer gene expression study, the cancer types may be based on histological classification of the tumor cells. Then, association tests, such as *F* tests, are conducted between the histological classification and transcriptional levels to discover genes of interest. However, the histological classification of cancer tumors may not distinguish important cancer subtypes (Alizadeh *et al.*, 2000; DeRisi *et al.*, 1996). This lack of information may lead to a spurious signal or reduced power in statistical inference.

When the external variables are unmeasured or imprecise, we are interested in using the latent variable basis, **L**, to discover genes of interest (Fig. 1). Because **L** is never directly measured, we must estimate it from the genomic data, using PCA and related methods. We apply our proposed method to two genomic datasets to demonstrate its utility in practice.

#### 4.2.1 Cell-cycle regulated gene expression in S. cerevisiae

It is known that in S. cerevisiae there is an abundance of genes whose transcription is regulated with respect to the cell cycle (Cho *et al.*, 1998; Spellman *et al.*, 1998). Nonetheless, comprehensive identification of the yeast genes whose expression is regulated by the cell cycle is still an active area of research, since it is unclear how the yeast cell-cycle regulation should be quantified and modeled (Pramila *et al.*, 2006; Rowicka *et al.*, 2007; Tu *et al.*, 2005; Wu and Li, 2008). The experimental time points after cell population synchronization are readily measured, but this external variable does not directly represent periodic transcriptional regulation with respect to the cell cycle.

Suppose that we want to carry out a hypothesis test on each gene of whether it shows regulation associated with a periodic pattern over the cell cycle. The null hypothesis is then that population mean is not periodic over the cell cycle. This null hypothesis contains an infinite number of mean time-course trajectories that are non-periodic, making the null hypothesis composite. A composite null hypothesis such as this one is largely intractable because it contains an unwieldy class of potential probability distributions describing gene expression. Indeed, a survey of the literature reveals that this composite null hypothesis is the major challenge when a traditional hypothesis testing approach is taken. However, using our approach, we can reduce the complexity of this problem by directly estimating the manifested systematic periodic expression variation and applying the proposed method to identify genes associated with this systematic variation due to the latent variables, **L**.

Spellman *et al.* (1998) measured transcriptional levels of *m* = 5981 yeast genes, every 30 min for 390 min after synchronizing the cell cycle among a population of cells by elutriation. The top two PCs capture the manifestation of cell-cycle regulation on gene expression (Alter *et al.*, 2000), explaining 48% of total variance (Fig. 2a, b). By testing for associations between time-course gene expression and the top two PCs, we avoid this challenging problem and consider instead the tractable association significance testing problem with a simple null hypothesis $H_{0}:\gamma_{i}=0$ versus $H_{1}:\gamma_{i}\neq 0$ (as opposed to a composite null). The hypothesis test is now simply whether gene *i* is associated with $\hat{r}=2$ latent variables estimated by the top two PCs.

We applied the proposed method (with *s* = 100 and B=2×m) to test this hypothesis and identified a large number of genes associated with yeast cell-cycle regulation. (We did not use functional PCA (Ramsay and Silverman, 2005; Yao *et al.*, 2005) to smooth the PCs with respect to time, although the jackstraw method is amendable to do so.) We discovered that approximately 84% of the 5981 measured genes are associated with the top two PCs (${\hat{\pi }}_{0}=0.16$). At FDR≤1%, 2998 genes were found to be statistically significant. Hierarchical clustering applied to these 2998 genes reveals the cell-cycle patterns captured by the top two PCs (Fig. 2c). The generalized proposed method allows us to compute statistical significance measures of associations with a subset of PCs. When testing for associations with the first PC while adjusting for the second PC, 1666 genes were called statistically significant at FDR≤1%, with the estimated proportion of null variables ${\hat{\pi }}_{0}=34.4\%$. On the other hand, at the same FDR threshold, we found 984 genes were significantly associated with the second PC with ${\hat{\pi }}_{0}=39.6\%$.

We applied the conventional test to the top two PCs in this data set and investigated its degree of over-fitting (yielding artificially small *P* values) as a function of the number of variables. This was accomplished by randomly sampling a subset of variables, applying each method to this subset of data, and then comparing the *P* value distributions of the jackstraw and conventional tests. It can be observed that smaller numbers of variables yield larger differences in the *P* value distributions, where the conventional test *P* values tend to be artificially small (Supplementary Fig. S8).

To explore the impact of the choice of *r* on the proposed method, we conducted the jackstraw analysis setting $\hat{r}=1$ and $\hat{r}=3$ in model (2) (Supplementary Fig. S10). Notably, we found that setting $\hat{r}=3$ yielded similar results to setting $\hat{r}=2$, similarly to what we observed in the simulation study (Supplementary Fig. S7). Setting $\hat{r}=1$ resulted in lower levels of statistical significance, and there was no obvious evidence of adverse effects from the fact that ignoring the 2nd PC induces dependence in the residuals of the model used with $\hat{r}=1$ (Leek and Storey, 2007, 2008).

It was demonstrated in the simulation studies that the proposed method produces valid null *P* values that satisfy the joint null criterion. To complement this analysis, we sought to verify on the real data set that applying the proposed algorithm with *s* = 100 and *B* = 10 produces *P* values that are similar to the most exhaustive method that makes the fewest assumptions. Specifically, we applied the proposed algorithm with *s* = 1 and *B* = 1000 where in calculating the *P* value for variable *i*, synthetic null statistics were constructed only on variable *i*. [The exhaustive method calculates within-gene *P* values, whereas the proposed method calculates *P* values from null statistics pooled across genes; see Leek and Storey (2011) for more on this distinction.] This required *B* = 1000 iterations of the algorithm for each of the *m* = 5981 genes, for a total of 5 981 000 SVD calculations and synthetic null statistics. Then, we calculated $p_{i}=\#\{F_{i}^{0b}\geq F_{i};b=1,\ldots ,1000\}/1000$ for each gene i=1,…,5981. This set-up gives an equivalent resolution to our proposed method with *s* = 100 and *B* = 10 because each *P* value is also based on 1000 synthetic null statistics. However, for the exhaustive method, the number of null statistic calculations is 5981-fold higher and the number of SVD calculations is 598 100-fold higher. We plotted the *P* values for each set-up against one another, where it can be seen in Supplementary Fig. S9 that the set of 5981 *P* values is very similar between the proposed method and the exhaustive method.

#### 4.2.2 Inflammation associated gene expression in post-trauma patients

Large-scale clinical genomic studies often lead to unique analytical challenges, including dealing with a large number of clinical variables, unclear clinical endpoints or disease labels, and expression heterogeneity (Leek and Storey, 2007). The ‘Inflammation and the Host Response to Injury’ (IHRI) consortium carried out a longitudinal clinical genomics study on blunt force trauma patients. They collected 393 clinical variables (some longitudinal) and time-course gene expression (total of 797 microarrays) on 168 post-trauma patients (Desai *et al.*, 2011). One of the main goals in this study was to elucidate how inflammatory responses after blunt force trauma are manifested on gene expression. To aggregate relevant clinical variables into a manageable daily score, the IHRI consortium used a modified version of the Marshall score to rate the severity of multiple organ dysfunction syndrome (Marshall *et al.*, 1995).

Based on the modified Marshall score trajectories, Desai *et al.* (2011) clustered post-trauma patients into five groups, called ‘ordered categorical Multiple Organ Failure’ (ocMOF) labels. The time-course gene expression profiles of each patient were summarized by ‘within patient expression changes’ (WPEC; Desai *et al.*, 2011). Then, they tested for correlations between the WPEC genomic variables and the ocMOF score to discover genes associated with inflammatory responses of post-trauma patients. However, the use of the potentially noisy ocMOF clinical variable may impose limitations, as patients with similar Marshall scores may exhibit a wide range of clinical outcomes (Cobb *et al.*, 2005). Furthermore, five discrete values for the ocMOF scores potentially limits the resolution of the clinical variable.

To investigate this further, we used our proposed approach where the gene expression itself was used to construct clinical phenotypes on the patients. We directly used the WPEC data to characterize the molecular signature of inflammatory responses to blunt force trauma. We estimated the manifestation of post-trauma inflammatory responses on gene expression, **L**, with the top nine PCs (Supplementary Fig. S11). Then, we applied the proposed method to identify the genomic variables in WPEC associated with the top nine PCs. The original analysis in Desai *et al.* (2011) estimated 24% of the 54 675 genomic variables (probe sets) to be associated with the ocMOF score. In contrast, our analysis revealed a much larger proportions of the genomic variables to be significantly associated with the major sources of variation, ranging from 62% for first PC to 39% for ninth PC.

The genes identified in the original analysis (Desai *et al.*, 2011) were largely identified in our analysis, although our analysis provided many more significant genes. To compare the biological relevance of our re-analysis versus the original analysis, we tested for enrichment of 17 inflammation-related gene sets (Loza *et al.*, 2007), using one-sided Mann–Whitney–Wilcoxon tests with permutation-based significance. At the FDR≤1%, none of the inflammatory-related gene sets is enriched for the original analysis using the ocMOF scores (Desai *et al.*, 2011). In contrast, a large number of inflammation-related gene sets are significantly enriched when the genomic variables are tested for the associations with the top nine PCs individually (Table 1). MAPK signaling is enriched for every PC, except fifth PC, whereas Innate Pathogen Detection is enriched for first, fourth, sixth, and ninth PCs, at the FDR≤1%. Those two biological pathways were emphasized in the original analysis (Desai *et al.*, 2011) as indicating down-regulation of innate pathogen detection and up-regulation of MAPK signaling pathway, and they were seen as strong predictors of long-term complications from brute force trauma. Based on enrichment tests, the proposed method appears to provide a biologically richer source of information than the analysis based on the ocMOF scores.

**Table 1.** *Q* values from gene enrichment analysis using inflammation-related gene sets

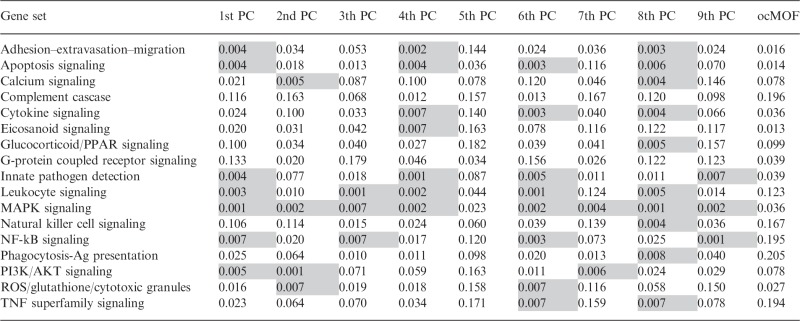

*Note* Darkened cells indicate *q* value ≤0.01 for a gene set enrichment test.

As with the previous study, we applied the conventional test in comparison to the jackstraw method as a function of number of variables, and we observed the same phenomenon where the conventional method clearly overfits as a function of the number of variables (Supplementary Fig. S8).

## 5 DISCUSSION

We have developed a method to accurately carry out statistical significance tests of associations between high-dimensional variables and latent variables, which have been estimated through systematic variation present in the observed high-dimensional variables themselves. Our approach is to maintain the overall systematic variation in the high-dimensional dataset, while replacing a small number of observed variables with independently permuted synthetic null variables. These synthetic null variables allow us to estimate the null distribution of the association statistics calculated on the original data that takes into account the inherent over-fitting that occurs when estimating latent variables through methods such as PCA. We call this approach the *jackstraw* because it draws on the idea of the game of jackstraws, where a player must remove a stick (i.e. a variable) from a pile of tangled sticks without disturbing the overall structure. Through extensive simulations, we demonstrated that the proposed method is capable of accounting for over-fitting and producing accurate statistical significance measures. We also demonstrated that applying conventional association testing methods to this problem artificially inflates the statistical significance of associations.

An input required for the proposed method is the number of PCs, *r*, that capture systematic variation from latent variables. Determining the number of ‘statistically significant’ PCs is an active area of research, and defining a number of significant PCs depends on the data structure and the context (Anderson, 1963; Buja and Eyuboglu, 1992; Johnstone, 2001; Leek, 2010; Tracy and Widom, 1996). Note that setting *r* to be too small leads to dependence in the residuals of model (2). This leads to the problems of dependence discussed in Leek and Storey (2007, 2008). Subsets of PCs can be considered while conditioning on other PCs in the jackstraw framework (Supplementary Material), so it is possible to avoid setting *r* to be too small. For example, if one would like to identify variables associated with the top three PCs, but is unsure whether the given data has three or four significant PCs, we have found it more robust to input $\hat{r}=4$, which will adjust for potential systematic residual variation captured by the fourth PC.

We demonstrated our approach using PCA. It is well known that individual PCs may not be directly interpretable or may contain multiple signals of interest that the user wishes to distinguish. The jackstraw method allows one to pinpoint a set of genomic variables associated with any given PC, a subset of PCs, a linear combination of two or more PCs, the projection of a subset of PCs onto an external variable, rotations of subsets of PCs, and low-dimensional latent variable estimates from other methods (see Supplementary Material). Therefore, this approach can be used to investigate and identify biological signals that may manifest in a particular subspace spanned by the estimated latent variables. We do not advocate blindly applying our method to the top *r* PCs without considering these issues.

Since the proposed method allows one to rigorously identify subsets of genomic variables associated with PCs, it allows one to also investigate whether these subsets have any biological coherence. This may be useful in investigating whether a space spanned by a subset of PCs captures relevant biological signal or is merely reflecting technical artifacts (e.g. batch effects in gene expression data). The method also improves the surrogate variable analysis algorithm of Leek and Storey (2007, 2008) in that it allows a more precise determination of the control variables that are used to estimate the surrogate variables. Thus, we have found the jackstraw to also be useful in the context of dealing with latent variables that reflect technical effects of no biological relevance.

The proposed method represents a novel resampling approach operating on variables, whereas established resampling approaches, such as the jackknife and the bootstrap, tend to operate on observations (Efron, 1979; Quenouille, 1949; Tukey, 1958). When applying these methods, systematic variation due to latent variables is intentionally perturbed, since their purpose is typically to assess the sampling variation of a single variable. In high-dimensional data, we may need to preserve systematic variation due to latent variables, which is the problem that the jackstraw addresses.

By accurately testing for associations between observed high-dimensional variables and the systematic manifestation of latent variables in the observed variables, our proposed method allows for the automatic discovery of complex sources of variation and the genomic variables that drive them. The proposed method extends PCA and related methods beyond their popular applications in exploring, visualizing and characterizing the systematic variation to genomic variable level (e.g. gene-level) significance analyses. Given the increasingly important role that non-parametric estimation of systematic variation plays in the analysis of genomic data (Alter *et al.*, 2000; Leek and Storey, 2007; Price *et al.*, 2006), the proposed method may be useful in many areas of quantitative biology using high-throughput technologies as well as other areas of high-dimensional data analysis.

## Supplementary Material

Supplementary Data
