# Supplementary material for: Statistical significance of variables driving systematic variation in high-dimensional data
Source: Bioinformatics. 2014 Oct 21;31(4):545–54. doi: 10.1093/bioinformatics/btu674 (PMC4325543; doi:10.1093/bioinformatics/btu674)
Supplement: Supplementary Data [file supp_31_4_545__index.html]

Statistical Significance of Variables Driving Systematic Variation in High-Dimensional Data — Statistical significance of variables driving systematic variation in high-dimensional data — Statistical significance of variables driving systematic variation in high-dimensional data — Supplementary Data 

# Statistical significance of variables driving systematic variation in high-dimensional data

## Supplementary Data

files

**Files in this Data Supplement:**

- Supplementary Data - pdf file
