# Supplementary material for: Statistical significance of variables driving systematic variation in high-dimensional data
Source: Bioinformatics. 2014 Oct 21;31(4):545–54. doi: 10.1093/bioinformatics/btu674 (PMC4325543; doi:10.1093/bioinformatics/btu674)
Supplement: Supplementary Data [file supp_btu674_supplementary.pdf]

Supporting Information: Statistical Significance of Variables Driving Systematic Variation in High-Dimensional Data

Neo Christopher Chung and John D. Storey

Generalization of the Proposed Algorithm to Subspaces Spanned by Principal Components.

Statistical Hypothesis Tests. Here, we expand the hypothesis test, $H_0 : \boldsymbol{\gamma}_i = \mathbf{0}$ vs. $H_1 : \boldsymbol{\gamma}_i \neq \mathbf{0}$, of model (2) to a more general test of a linear hypothesis. Let Ω_0 be the null space of interest and Ω_1 the alternative space. Suppose that $\mathbf{C}$ is a $r \times q$ matrix where $r \geq q$ and $\boldsymbol{a}$ is a q-vector. Then $\Omega_0 = \{\boldsymbol{\gamma} \in \mathbb{R}^r : \boldsymbol{\gamma}\mathbf{C} = \boldsymbol{a}\}$ and $\Omega_1 = \{\boldsymbol{\gamma} \in \mathbb{R}^r : \boldsymbol{\gamma}\mathbf{C} \neq \boldsymbol{a}\}$ is a general representation for a null and an alternative space, respectively, of a test of a linear hypothesis. See Draper and Smith (1998) for a thorough treatment of tests of linear hypotheses.

The hypothesis test $H_0 : \boldsymbol{\gamma}_i = \mathbf{0}$ vs. $H_1 : \boldsymbol{\gamma}_i \neq \mathbf{0}$ for each gene i applied to model (2) can be generalized to

$$H_0 : \boldsymbol{\gamma}_i \in \Omega_0$$
$$H_1 : \boldsymbol{\gamma}_i \in \Omega_1.$$

The proposed algorithm can be modified so that the regression based association test performs this hypothesis test (Steps 2 and 5 in the main text algorithm). This is straightforwardly done by forming F-statistics that are calculated by comparing the unconstrained model fit of model (2) to that under the constraint $\boldsymbol{\gamma}_i \in \Omega_0$ (see Draper and Smith, 1998).

One important procedure enabled by this generalization is to identify variables associated with a subset of rows of $\mathbf{V}_r^T$. Among multiple statistically significant PCs ($r \geq 2$), there may be a subset of PCs of interest, denoted by $\mathbf{V}_{r_1}^T$. The complementary subset of PCs that are not of interest is denoted by $\mathbf{V}_{r_0}^T$. The proposed method allows one to estimate the significance of associations between observed variables and $\mathbf{V}_{r_1}^T$, while adjusting for $\mathbf{V}_{r_0}^T$. Operationally, $\mathbf{V}_{r_0}^T$ has to be included in both the constrained and unconstrained models when calculating the F-statistics, whereas $\mathbf{V}_{r_1}^T$ is only included in the unconstrained model fit. As illustrated in the simulation study, suppose that $r = 2$ and we are interested in identifying observed variables associated only with the 1st PC. In this case, $\Omega_0 = \{(0, \gamma_{i,2}) : \gamma_{i,2} \in \mathbb{R}\}$ and $\Omega_1 = \{(\gamma_{i,1}, \gamma_{i,2}) : \gamma_{i,1} \neq 0 \text{ and } \gamma_{i,1}, \gamma_{i,2} \in \mathbb{R}\}$. In the proposed algorithm, the 2nd PC ($= \mathbf{V}_{r_0}^T$)

has to be included in both the constrained and unconstrained models when calculating the F-statistics, whereas the 1st PC ($= \mathbf{V}_{r_1}^T$) is only included in the unconstrained model fit. This scenario with two PCs was simulated, and we demonstrated that the proposed algorithm can produce valid p-values, while accounting for 2nd PC (Fig. S6). As expected, the conventional F-test results in artificial inflation of statistical significance.

Rotations of Principal Components. The top r PCs, $\mathbf{V}_r^T$, collectively capture the systematic variation and estimate the row spanned by $\mathbf{L}(\mathbf{z})$ (Leek, 2010). $\mathbf{V}_r^T$ may be rotated while spanning the same row space. The proposed method is capable of estimating the statistical significance of associations between observed variables and any rotation of $\mathbf{V}_r^T$. Consider a $r \times r$ rotation matrix, $\mathbf{R}$. Then, the generalization of model (2) is

$$\begin{aligned} \mathbf{Y} &= \boldsymbol{\Gamma}\mathbf{R}\mathbf{V}_r^T + \mathbf{E}' \\ &= \boldsymbol{\Gamma}\mathbf{W}_r + \mathbf{E}'. \end{aligned} \tag{3}$$

To perform a significance test of $H_0 : \boldsymbol{\gamma}_i \in \Omega_0$ vs. $H_1 : \boldsymbol{\gamma}_i \in \Omega_1$ for each gene i, the algorithm needs to adapt the model (3) with $\mathbf{R}\mathbf{V}_r^T$, in place of model (2) with $\mathbf{V}_r^T$. The rotation matrix $\mathbf{R}$ must be applied on $\mathbf{V}_r^{*T}$ in every iteration of the estimation step of null association statistics. This generalization allows one to perform hypothesis tests using various rotations and projections of PCs.

The rotation matrix $\mathbf{R}$ could reflect biological or clinical measurements, independent of genomic data. By regressing the top r PCs on a noisy phenotype, we may improve the molecular signature of interest (e.g., separating technical artifacts from biological variation). For example, let's assume a high-dimensional genomic data set contains three statistically significant PCs ($r = 3$) and we are interested in identifying variables associated with a particular linear combination of the top three PCs: $\mathbf{w}_1 = 0.5\mathbf{v}_1^T - 0.5\mathbf{v}_2^T + \sqrt{0.5}\mathbf{v}_3^T$. Then, we may construct a 3×3 rotation matrix $\mathbf{R}$, which must be orthonormal whose determinant equals 1, as required by any proper rotation matrix. In this case, we arrive at

$$\mathbf{R} = \begin{bmatrix} 0.5 & -0.5 & \sqrt{0.5} \\ 0.5 & -0.5 & -\sqrt{0.5} \\ \sqrt{0.5} & \sqrt{0.5} & 0 \end{bmatrix}.$$

After obtaining this particular rotation of the top three PCs, $\mathbf{W}_r = \mathbf{R}\mathbf{V}_r^T$, we can continue onto performing a significance test using model (3). The two modifications to the algorithm are:

1. To apply $\mathbf{R}$ on both $\mathbf{V}_r^T$ and $\mathbf{V}_r^{*T}$ to obtain $\mathbf{W}_r = \mathbf{R}\mathbf{V}_r^T$ and $\mathbf{W}_r^* = \mathbf{R}\mathbf{V}_r^{*T}$ in the computation of observed association statistics and null association statistics, respectively.

2. To construct a hypothesis test of association between variable i and $\mathbf{w}_1$, while accounting for $\mathbf{w}_2$ and $\mathbf{w}_3$: $\Omega_0 = \{(0, \gamma_{i,2}, \gamma_{i,3}) : \gamma_{i,2}, \gamma_{i,3} \in \mathbb{R}\}$ and $\Omega_1 = \{(\gamma_{i,1}, \gamma_{i,2}, \gamma_{i,3}) : \gamma_{i,1} \neq 0 \text{ and } \gamma_{i,1}, \gamma_{i,2}, \gamma_{i,3} \in \mathbb{R}\}$.

Another interesting example of this generalized jackstraw algorithm arises from transformations of $\mathbf{V}_r^T$ based on a statistical criterion. Starting with $\mathbf{V}_r^T$, Independent Component Analysis (ICA) seeks r components that are mutually statistically independent (Comon, 1994). In some cases with statistically independent and non-Gaussian sources of variation, independent components may be more sensible, providing an interpretable low-dimensional representation. We may obtain independent components by rotating $\mathbf{V}_r^T$ to maximize mutual statistical independence (Hastie et al., 2011). Let $\mathbf{R}$ be a rotation matrix that maps $\mathbf{V}_r^T$ into the r independent components. To carry out the association test on the r independent components, we use the generalized model (3) with $\mathbf{R}\mathbf{V}_r^T$, instead of model (2) with $\mathbf{V}_r^T$. As above, the analogous substitution occurs at every iteration to ensure the same rotation is applied to synthetic null variables.

Generalized Algorithm to Calculate Significance of Variables Associated with PCs

1. Obtain r PCs of interest, $\mathbf{V}_r^T$ by applying SVD to the row-centered matrix $\mathbf{Y}_{m \times n} = \mathbf{U}\mathbf{D}\mathbf{V}^T$.
2. Rotate $\mathbf{V}_r^T$ using a $r \times r$ rotation matrix $\mathbf{R}$ to model $\mathbf{Y} = \mathbf{\Gamma}\mathbf{R}\mathbf{V}_r^T + \mathbf{E}'$.
3. Calculate m observed F-statistics $F_1, \ldots, F_m$, testing $H_0 : \boldsymbol{\gamma}_i \in \Omega_0$ vs. $H_1 : \boldsymbol{\gamma}_i \in \Omega_1$ from the Step 2 model.
4. Randomly select and permute s rows of $\mathbf{Y}_{m \times n}$, resulting in $\mathbf{Y}^*_{m \times n}$.
5. Obtain $\mathbf{V}_r^{*T}$ from SVD applied to $\mathbf{Y}^* = \mathbf{U}^*\mathbf{D}^*\mathbf{V}^{*T}$.
6. Rotate $\mathbf{V}_r^{*T}$ using a $r \times r$ rotation matrix $\mathbf{R}$ to model $\mathbf{Y}^* = \mathbf{\Gamma}^*\mathbf{R}\mathbf{V}_r^{*T} + \mathbf{E}'^*$.
7. Calculate null F-statistics $F_1^{0b}, \ldots, F_s^{0b}$ from the s synthetic null rows of $\mathbf{Y}^*$, testing $H_0 : \boldsymbol{\gamma}_i^* \in \Omega_0$ vs. $H_1 : \boldsymbol{\gamma}_i^* \in \Omega_1$ from the Step 6 model.
8. Repeat steps 4-7 for $b = 1, \ldots, B$ iterations to obtain a total $s \times B$ of null F-statistics.
9. Compute the p-value for variable i ($i = 1, \ldots, m$) by:
$$p_i = \frac{\#\{F_j^{0b} \geq F_i; j = 1, \ldots, s, b = 1, \ldots, B\}}{s \times B}$$
10. Identify statistically significant tests based on the p-values $p_1, p_2, \ldots, p_m$ (e.g., using false discovery rates).

Remark 1. If one wants to test linear hypotheses on the unrotated PCs, then Step 2 is the above algorithm is carried out such that $\mathbf{R}$ is the identity matrix.

Remark 2. Step 4 of the generalized algorithm above permutes s rows of $\mathbf{Y}$, thereby breaking all systematic variation spanned by $\mathbf{V}_r^T$ among these s variables. If one is testing for associations on only a subspace of $\mathbf{V}_r^T$, then it may be desirable to preserve the systematic variation spanned by the subspace of $\mathbf{V}_r^T$ acting as adjustment variables. (E.g., If one is testing for associations with the first PC where $r = 2$, then it may be desirable to preserve the systematic variation spanned by the second PC among these s variables.) This can be accomplished, for example, by modifying the permutations to act only on the residuals after regressing out the adjustment variables, or by carrying out a bootstrap null procedure whereby residuals resulting from this regression are bootstrapped and added back to the adjustment variable only model fit (see, e.g., Efron and Tibshirani (1993)).

An alternative delete-s approach.

We also investigated a delete-s version of the jackstraw, which draws on ideas from our proposed method, the permute-s jackstraw. In the delete-s jackstraw, the set of m variables is broken up into m/s disjoint sets of s variables each, where $s \ll m$. The significance of associations between a given set of s variables and unobserved latent variables is then calculated by testing for the associations between each of the s variables and the top r PCs calculated among the remaining $m - s$ variables. Since the set of s variables are not used to estimate the latent variables, the association p-values for those s variables should be marginally valid. However, we found that the delete-s jackstraw did not satisfy the joint null criterion. We applied the delete-s jackstraw to the above 16 simulation scenarios and found that the delete-s jackstraw results in an anti-conservative bias (Fig. S12). As s increases, the null p-values from the delete-s jackstraw exhibited a greater anti-conservative bias. Since s variables are deleted for each set, the PCs for a given set constructed based on $m-s$ variables share $m - 2s$ variables with the PCs for any other set. We hypothesize that this delete-s approach did not produce valid null p-values because of a complex dependence among the m/s sets of s p-values, similar to dependence issues encountered in cross-validation.

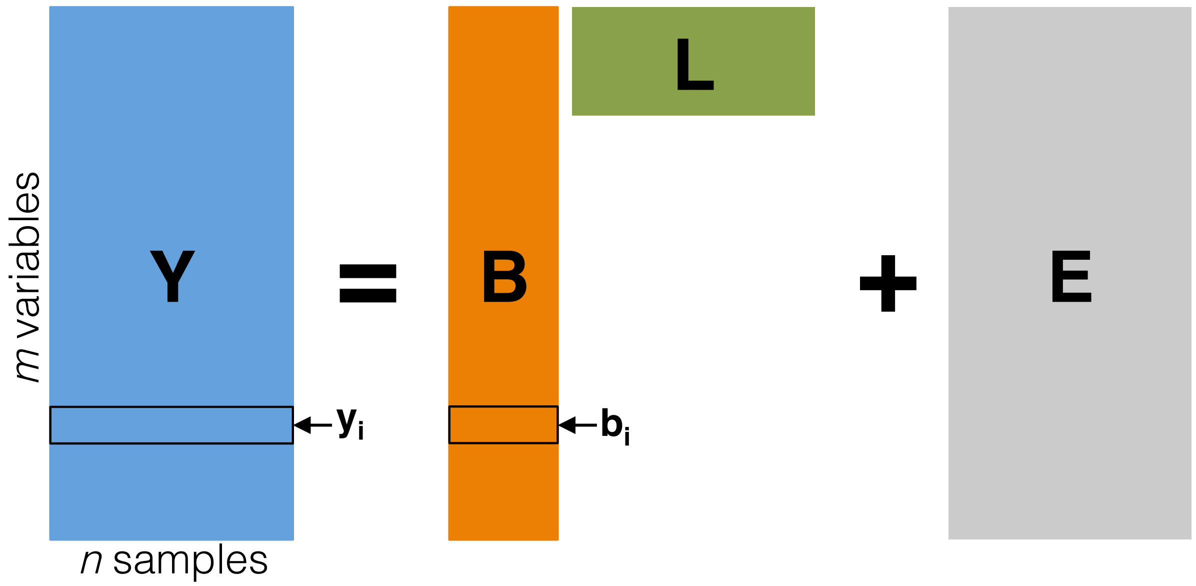

Figure S1: Diagram of the latent variable model (1). The latent variable basis $\mathbf{L}$ is not observable, but may be estimated from $\mathbf{Y}$ using the top r right singular vectors ($\mathbf{V}_r^T$). The noise term $\mathbf{E}$ is independent random variation. We are interested in performing statistical hypothesis tests on $\mathbf{b}_i$ ($i = 1, \ldots, m$), which quantifies the relationship between $\mathbf{L}$ and $\mathbf{y}_i$ ($i = 1, \ldots, m$). Since $\mathbf{L}$ must be estimated from $\mathbf{Y}$, a conventional association test results in anti-conservative p-values. We have developed the jackstraw method to account for over-fitting due to using estimates of $\mathbf{L}$ to compute the statistical significance of associations between $\mathbf{L}$ and $\mathbf{y}_i$.

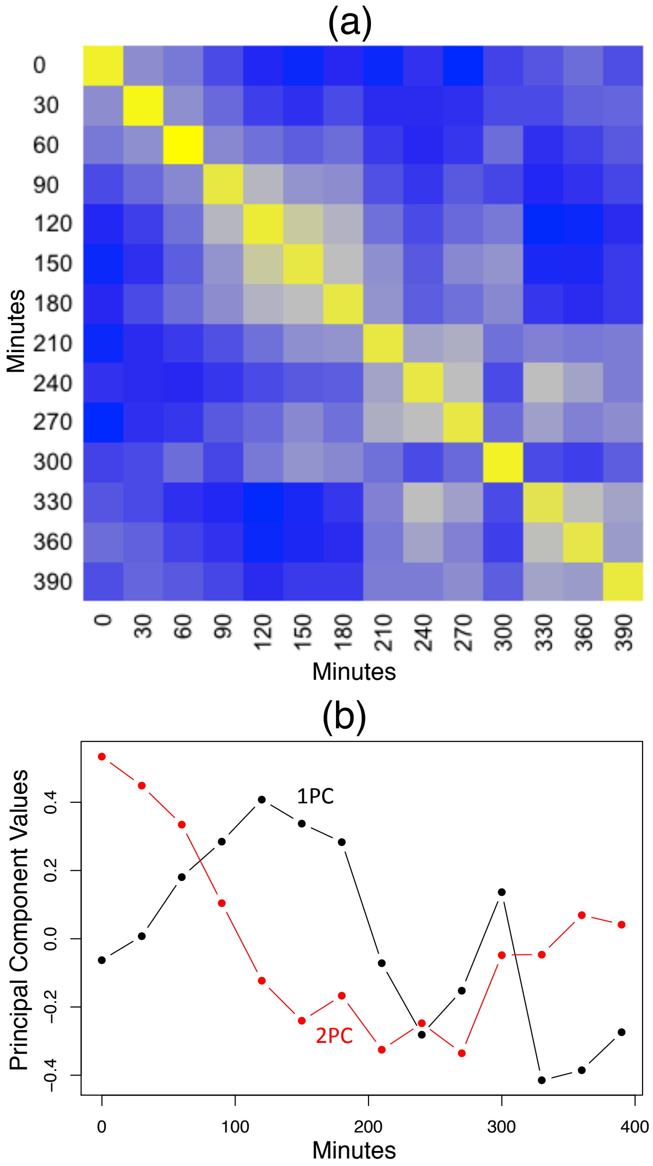

Figure S2: Original gene expression profiles of the Spellman *et al.* (1998) yeast cell cycle experiment. (a) A heat map of the covariance matrix of 14 arrays reveals an outlier from the time point at 300 min (low values in blue and high values in yellow). (b) The top two PCs of the original 14 microarrays corroborate an aberrant gene expression profile from 300 min, which is removed from our new analysis.

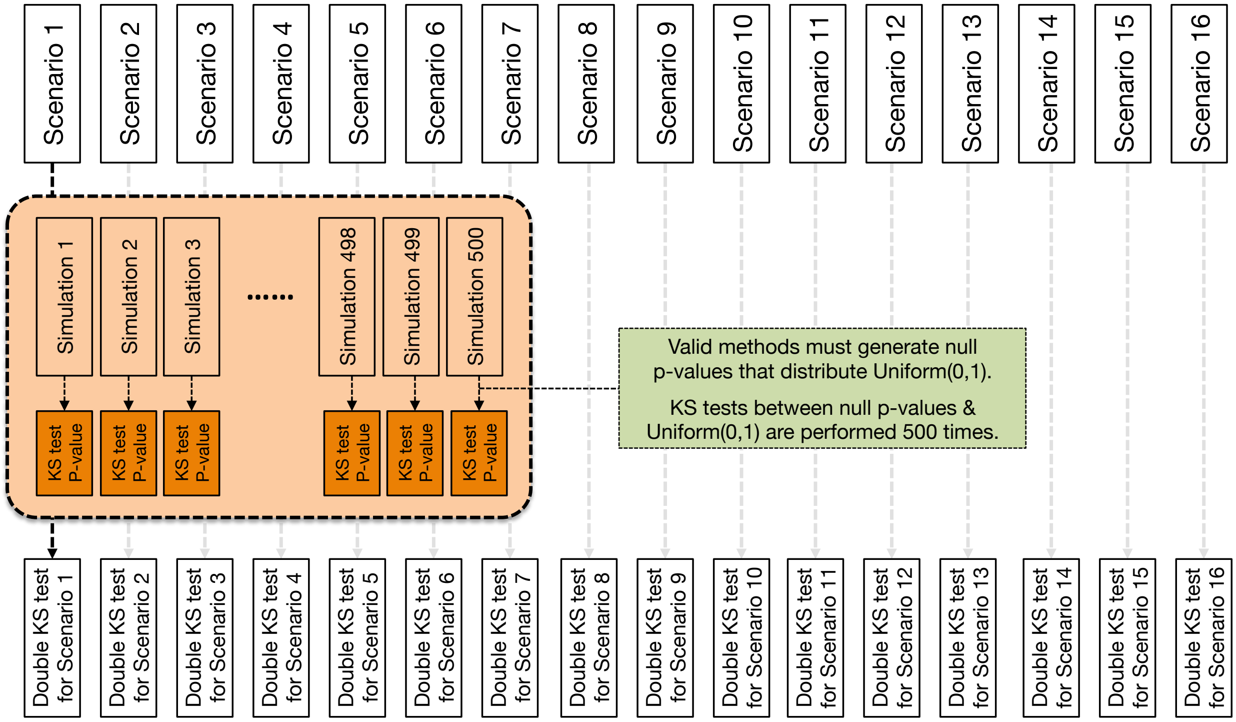

Figure S3: Evaluation pipeline for 16 simulation scenarios. To assess statistical accuracy in testing the associations between variables and principal components, we generated 16 scenarios from a wide range of configurations. For a given scenario, we simulated 500 independent studies, which resulted in 500 KS test p-values (refer to a green box). For a valid statistical method, this set of 500 KS test p-values should be distributed Uniform(0,1); we evaluate an anti-conservative bias among 500 KS test p-values with another application of the KS test (a "double KS test") . This results in the 16 simulation scenarios being summarized by 16 double KS test p-values (Fig. 6), giving us a comprehensive view of the method's statistical accuracy.

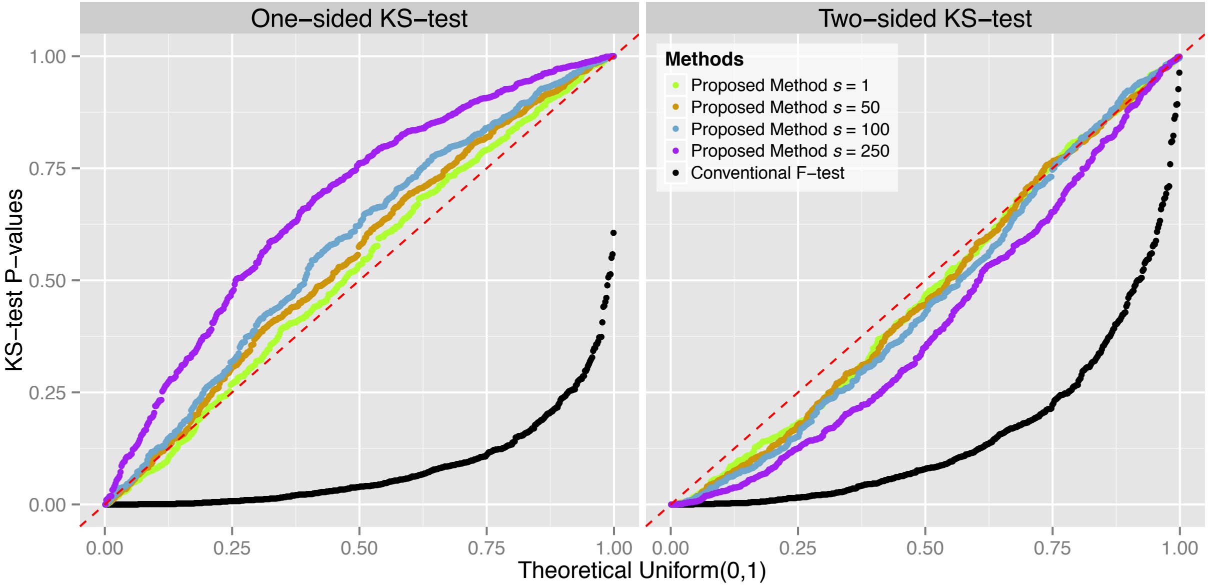

Figure S4: QQ-plots of KS test p-values from the highlighted simulation scenario in the main text, using the conventional F-test and the proposed method. In this scenario, we generated a dichotomous mean shift between two groups of observations with the true proportion of null variables $\pi_0 = 0.95$. We assessed the Uniform(0,1) property of null p-values using the Kolmogorov-Smirnov (KS) test. In the left QQ-plot comparing one-sided KS test p-values and the Uniform(0,1) distribution, the downward displacement of the black points below the diagonal red dashed line indicates anti-conservative p-values resulting from the conventional F-test. In contrast, the upward displacement of the colored points demonstrates how the proposed method guards against anti-conservative bias. On the right panel, a two-sided KS test detects any deviation – both conservative and anti-conservative – from the theoretically correct Uniform(0,1) distribution.

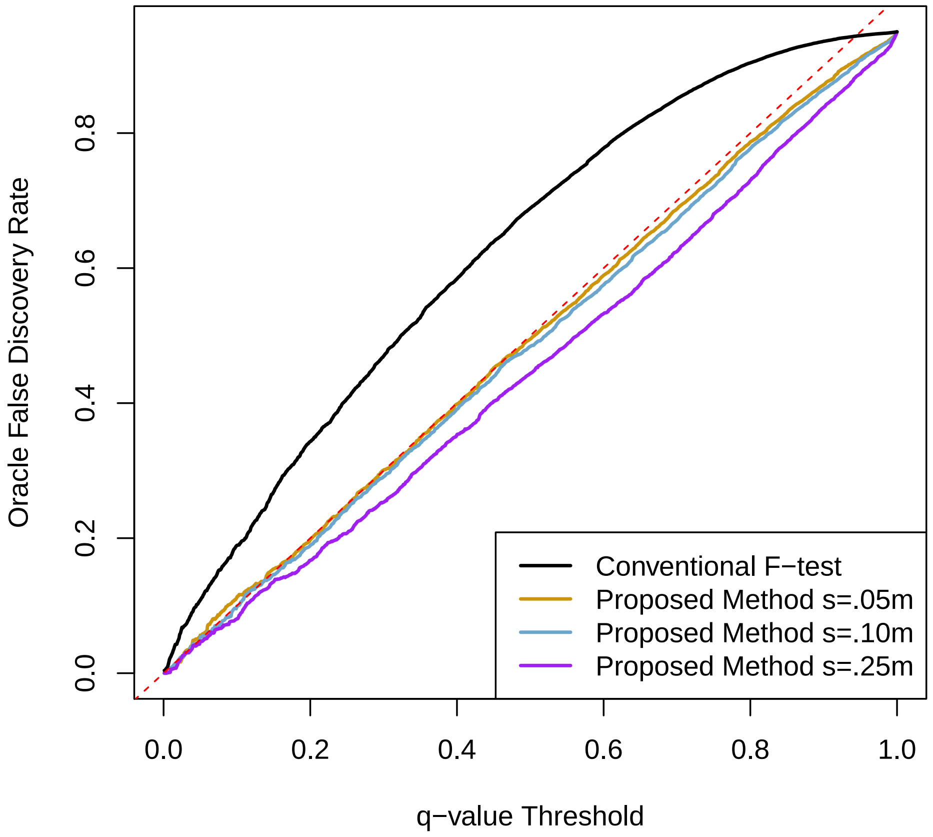

Figure S5: False discovery rate (FDR) estimation comparison between the conventional F-test and the proposed method. Q-values are estimated for 500 independent simulation studies from the highlighted simulation scenario. Estimated FDRs from the conventional F-test demonstrate an anti-conservative bias, which deviates upward from a theoretically correct diagonal line (red dashed). In contrast, the proposed jackstraw properly controls FDR, while exhibiting a conservative bias with an increasing value of s.

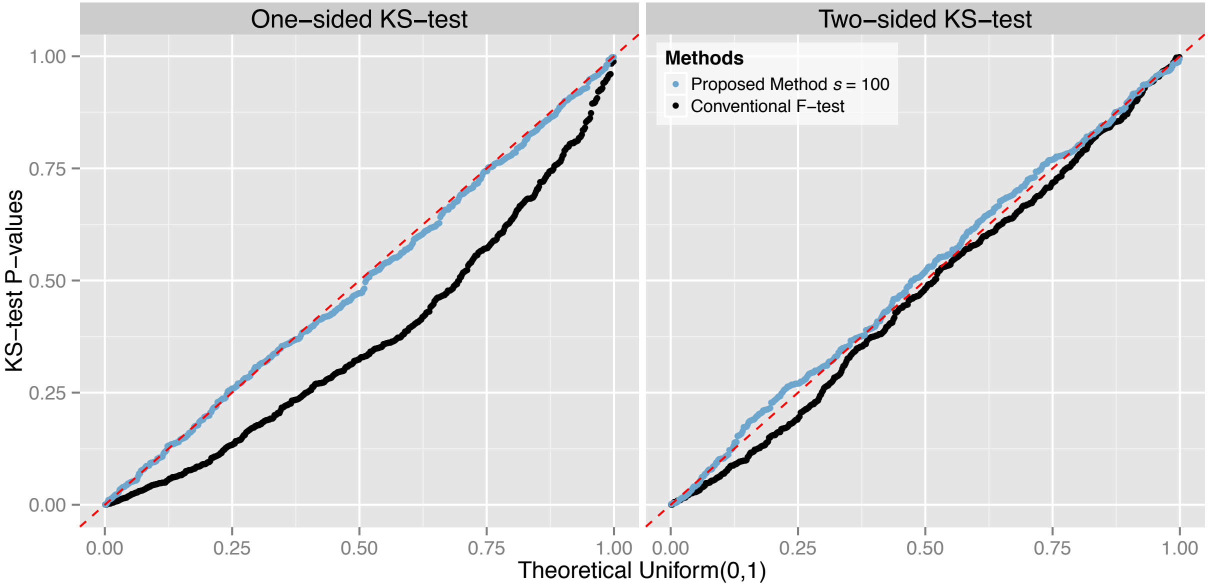

Figure S6: Evaluation of statistical tests for the associations on subsets of principal components (PCs). We simulated $m = 1000$ variables of $n = 20$ observations, with $r = 2$ significant PCs. We applied the conventional F-test and the proposed method to test for associations between m variables and the 1st PC. The proposed method is capable of correctly estimating significance measures of the associations between variables and their 1st PC, while adjusting for the 2nd PC. The conventional F-test produces anti-conservative p-values due to overfitting, where KS test p-values are skewed towards 0. See *Testing for associations on subsets of principal components* in Section 4 for a description of this simulation scenario.

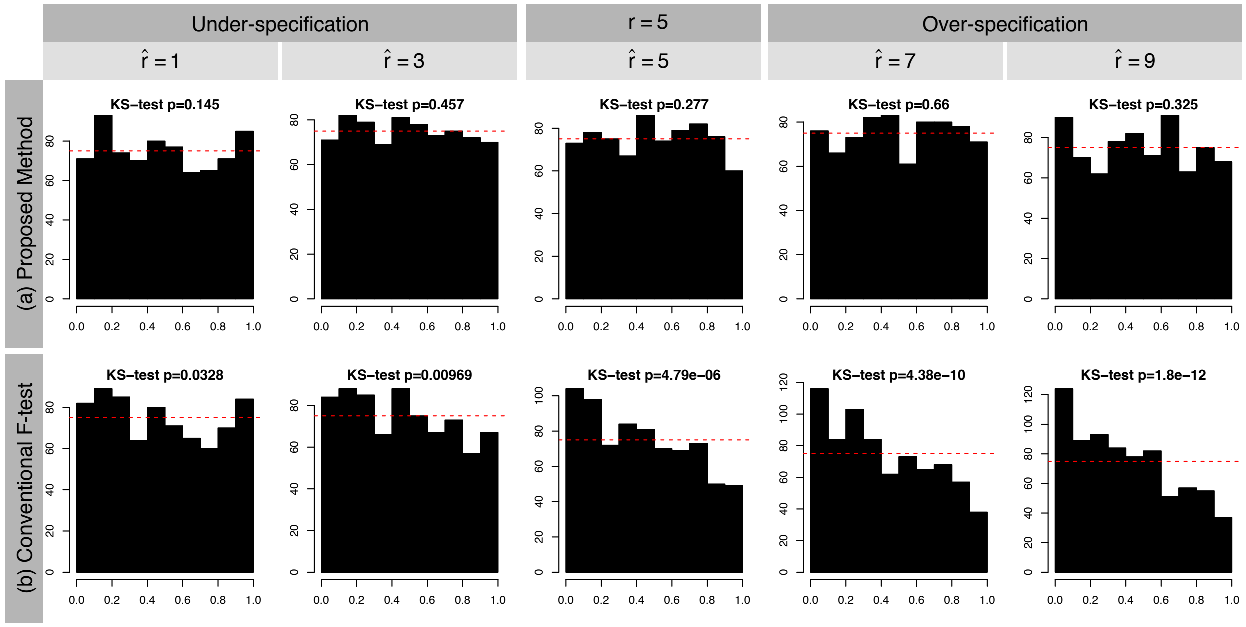

Figure S7: Impact of misspecification of r on the proposed jackstraw and conventional F-test. For $m = 1000$ variables of $n = 20$ observations ($\pi_0 = 0.75$), we simulated $r = 5$ latent variables simulated from one of each of the following distributions: a randomized dichotomous variable, Normal(0,1), Uniform(0,1), Bin(2, 0.5), and Normal(0,0.25). We applied the jackstraw algorithm with $s = 0.1m$ and the conventional F-test to the simulated data with $\hat{r} = 1, 3, 5, 7, 9$ used in model (2). To detect an anti-conservative bias, we applied a one-sided KS-test on p-values corresponding to the true null variables. Since there exist in truth $r = 5$ latent variables, the results with $\hat{r} = 1, 3$ and $\hat{r} = 7, 9$ demonstrate the operating characteristics when the number of PCs is under- or over-specified, respectively.

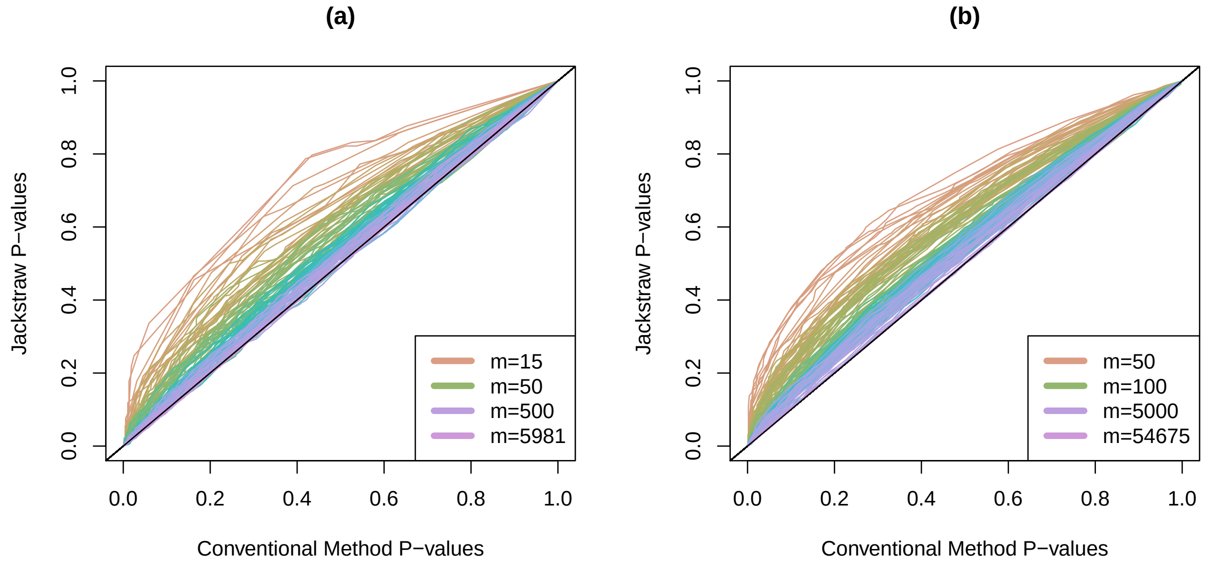

Figure S8: QQ-plots of p-values from the proposed jackstraw method and conventional F-test applied on *(a)* the yeast cell-cycle gene expression data from Spellman *et al.* (1998) and *(b)* the post-trauma Within Patient Expression Change matrix from Desai *et al.* (2011). Over a range of number of variables m, we randomly selected a subset of variables from the original data sets and performed a full analysis on the data subset using each method. A QQ-plot of the p-values from each method is plotted for each value of m. Generally, the unadjusted conventional p-values (x-axis) are smaller than the jackstraw p-values (y-axis), demonstrating that the conventional method tends to produce artificially small p-values. The plots shows that smaller m tends to yield larger differences between the jackstraw and conventional methods.

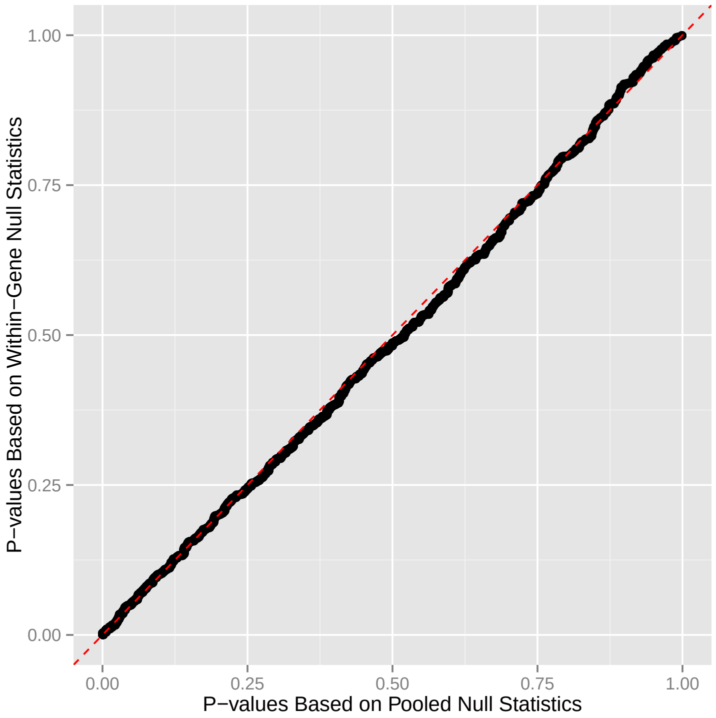

Figure S9: QQ-plots of p-values from the proposed method (x-axis) versus p-values based on null statistics generated separately for each gene (y-axis). By default, the jackstraw pools synthetic null statistics across all variables under consideration; in this case, we ran the jackstraw with $s = 100$ and $B = 10$ (to obtain 1000 null statistics) on the Spellman *et al.* (1998) data. In contrast, one may compute 1000 null statistics for each of 5981 yeast genes in the dataset, and a p-value for a given gene is computed using only the 1000 null statistics generated from that gene. This exhaustive method is described in the main text.

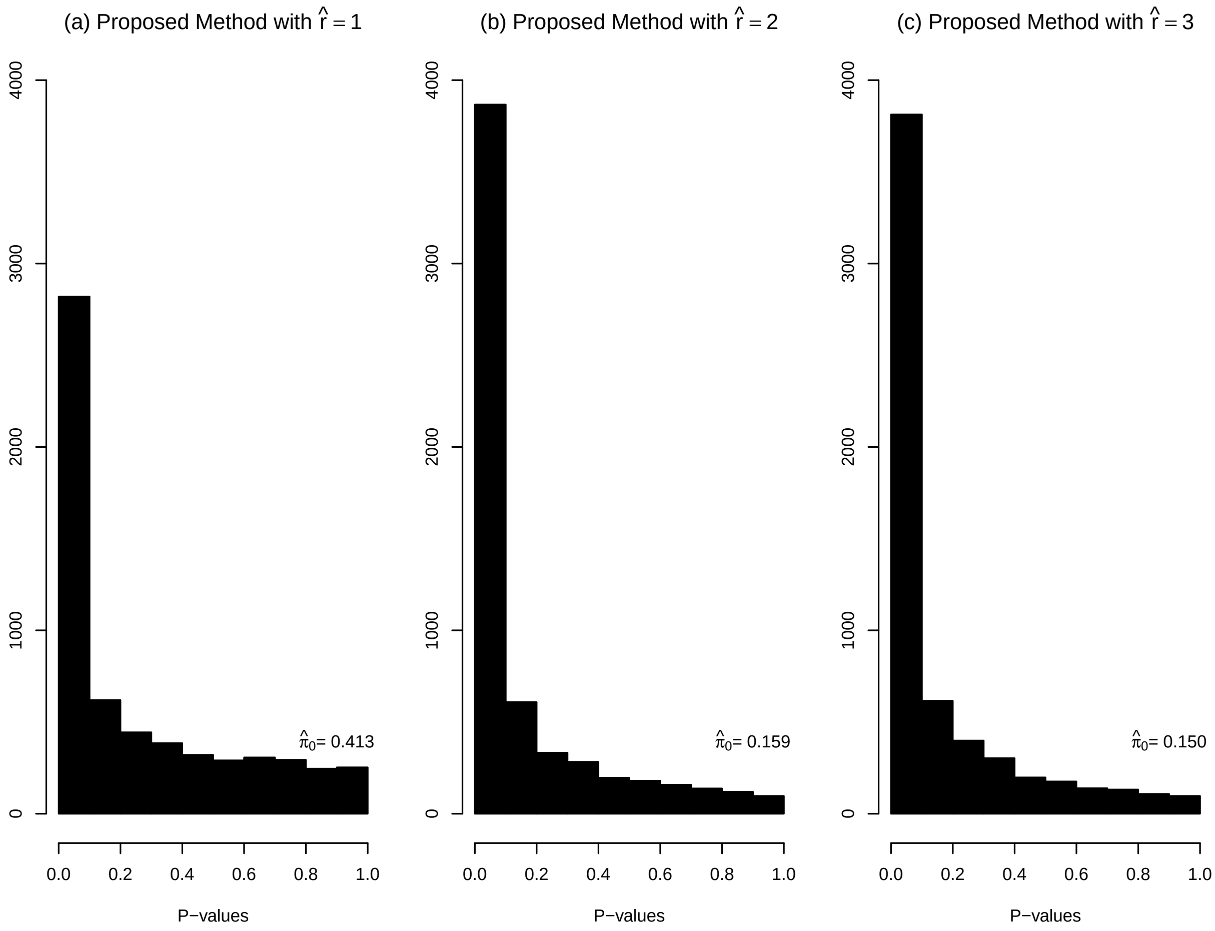

Figure S10: Effect of misspecification of r on gene expression profiles of the Spellman *et al.* (1998) yeast cell cycle experiment. The proposed jackstraw method requires a user to estimate and input the number of significant PCs r. While Alter *et al.* 2001 suggests $\hat{r} = 2$ for this particular dataset, we also ran the jackstraw algorithm with under-specified $\hat{r} = 1$ and over-specified $\hat{r} = 3$.

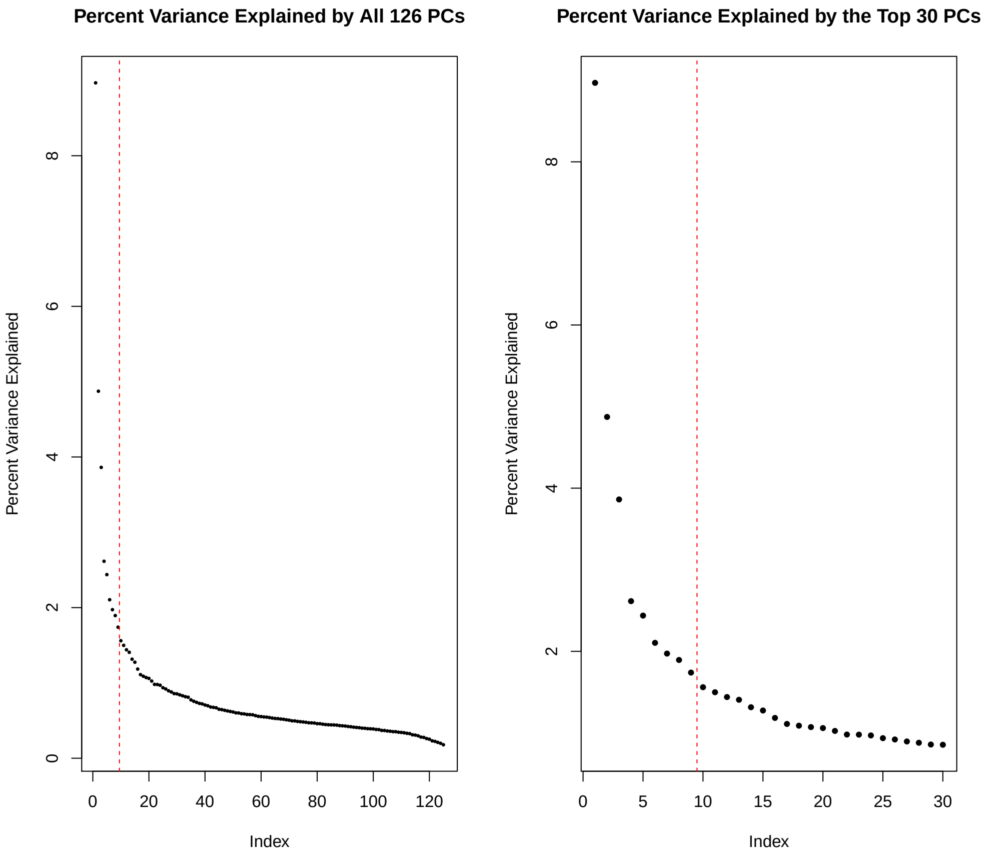

Figure S11: Percent variance explained by principal components (PCs) of the "Within Patient Expression Change (WPEC)" matrix from Desai *et al.* (2011). The considerable drop (the "elbow") in this scree plot helped us to identify the top 9 PCs as significant, likely capturing the molecular signatures of patient responses to blunt force trauma.

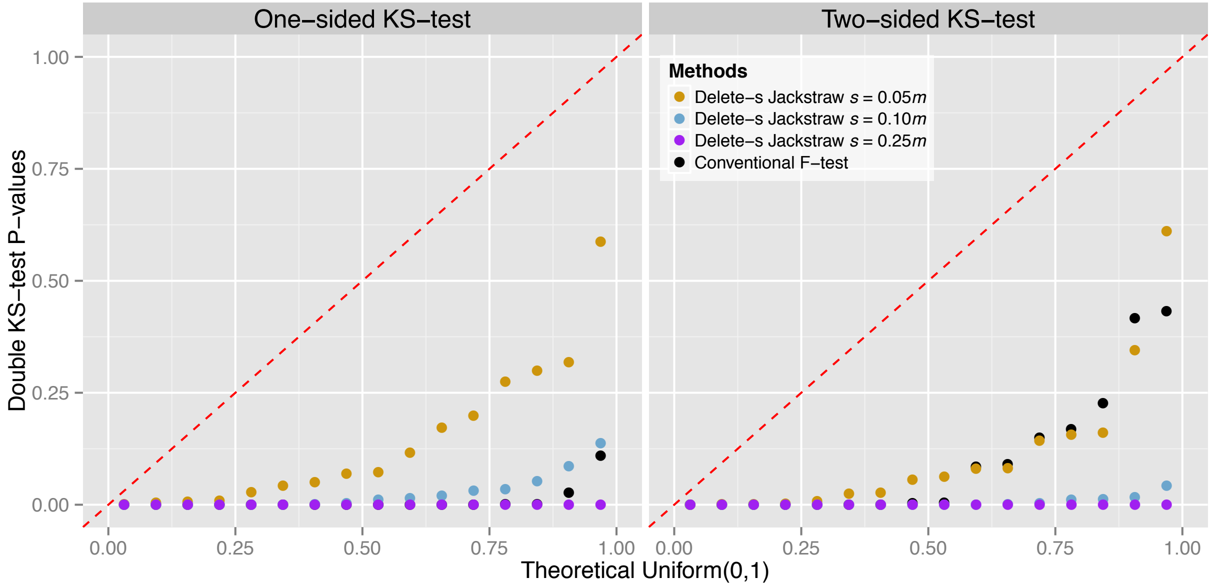

Figure S12: QQ-plots of double KS-test p-values from applying the delete-s jackstraw on 16 simulation scenarios. In a delete-s version of the jackstraw, the association p-values between the latent variable basis and a given set of s variables are estimated by testing for the associations between the PCs from $m - s$ variables and the separate set of s variables, which were left out in computation of the PCs. A systematic downward displacement of the points below the red diagonal line indicates anti-conservative p-values, present in both the conventional F-test and the delete-s jackstraw method. A larger value of s in the delete-s jackstraw leads to a greater anti-conservative bias.

References

Draper, N. R. and Smith, H. (1998). *Applied Regression Analysis*, 3rd edn, Wiley Series in Probability and Statistics.

Efron, B. and Tibshirani, R. J. (1993). *An Introduction to the Bootstrap*, Chapman & Hall.
